# Saxagliptin but Not Sitagliptin Inhibits CaMKII and PKC via DPP9 Inhibition in Cardiomyocytes

**DOI:** 10.3389/fphys.2018.01622

**Published:** 2018-11-14

**Authors:** Chintan N. Koyani, Christopher Trummer, Niroj Shrestha, Susanne Scheruebel, Benjamin Bourgeois, Ioanna Plastira, Sandra Kickmaier, Harald Sourij, Peter P. Rainer, Tobias Madl, Wolfgang Sattler, Brigitte Pelzmann, Ernst Malle, Dirk von Lewinski

**Affiliations:** ^1^Division of Cardiology, Medical University of Graz, Graz, Austria; ^2^Molecular Biology and Biochemistry, Gottfried Schatz Research Center, Medical University of Graz, Graz, Austria; ^3^Biophysics, Gottfried Schatz Research Center, Medical University of Graz, Graz, Austria; ^4^Division of Endocrinology and Diabetology, Department of Internal Medicine, Medical University of Graz, Graz, Austria; ^5^Center for Biomarker Research in Medicine, Graz, Austria; ^6^BioTechMed-Graz, Graz, Austria

**Keywords:** diabetes, heart failure, gliptins, Ca^2+^ transient, cardiac electrophysiology

## Abstract

Some oral anti-hyperglycemic drugs, including gliptins that inhibit dipeptidyl peptidase 4 (DPP4), have been linked to the increased risk of heart failure (HF) in type-2 diabetic patients. While the cardiovascular safety trial, TECOS, revealed no link between sitagliptin and the risk of HF, a substantial 27% increase in the hospitalization for HF was observed in type-2 diabetic patients treated with saxagliptin within the SAVOR-TIMI 53 trial. A previous *in vitro* study revealed that saxagliptin impairs the Ca^2+^/calmodulin-dependent protein kinase II (CaMKII)-phospholamban (PLB)-sarcoplasmic reticulum Ca^2+^-ATPase 2a axis and protein kinase C (PKC) activity in cardiomyocytes leading to impaired cardiac contractility and electrophysiological function. However, the link between saxagliptin and its target proteins (CaMKII and PKC) remains to be explored. Since DPP8 and DPP9 (but not DPP4) are expressed by cardiomyocytes and saxagliptin is internalized by cardiomyocytes, we investigated whether DPP8/9 contribute to saxagliptin-mediated inhibition of CaMKII and PKC activity. Structural analysis revealed that the DPP4-saxagliptin interaction motif (S630, Y547) for the cyanopyrrolidine group is conserved in DPP8 (S755, Y669) and DPP9 (S730, Y644). Conversely, F357 that facilitates binding of the anchor lock domain of sitagliptin in the S2 extensive subsite of DPP4 is not conserved in DPP8/9. In parallel, unlike saxagliptin, sitagliptin did not affect phosphorylation of CaMKII/PLB or activity of PKC in HL-1 cardiomyocytes. These findings were recapitulated by pharmacological inhibition (TC-E-5007, a DPP8/9 antagonist) and knock-down of DPP9 (but not DPP8). In primary mouse ventricular cardiomyocytes, saxagliptin (but not sitagliptin) impaired Ca^2+^ transient relaxation and prolonged action potential duration (APD). These results suggest that saxagliptin-DPP9 interaction impairs the CaMKII-PLB and PKC signaling in cardiomyocytes. We reveal a novel and potential role of DPP9 in cardiac signaling. The interaction of saxagliptin with DPP9 may represent an underlying mechanism for the link between saxagliptin and HF. Elucidation of saxagliptin-DPP9 interaction and downstream events may foster a better understanding of the role of gliptins as modulators of cardiac signaling.

## Introduction

Prevalence of diabetes mellitus (DM) is increasing worldwide and about 90% of all DM patients suffer from type 2 DM (T2DM). Hyperglycemia induces micro- and macro-vascular complications resulting in cardiac, kidney, eye and vessel dysfunction (Peter et al., 2008). One of the major causes of T2DM-associated mortality is heart failure (HF) (McMurray et al., 2014), though the exact underlying pathophysiological events responsible for DM-induced HF are still not clearly understood.

Both, HF and DM are multifactorial diseases that share some common etiological and risk factors including imbalanced ionic homeostasis, ion channel dysfunction, oxidative stress and aberrant metabolic homeostasis (Miki et al., 2013; Kasznicki and Drzewoski, 2014). Diabetic cardiomyopathy leads to impaired cardiac relaxation and systolic and/or diastolic dysfunction that are observed also in failing hearts (Bleske, 2000; Mandinov et al., 2000). Moreover, prolongation of cardiac action potential duration (APD) is often observed during chronic DM and HF (Wang and Hill, 2010).

Apart from DM being a predisposing factor for the development of HF, therapeutic remedies of DM are linked to the risk of HF as well (Udell et al., 2015). Anti-diabetic drugs, including thiazolidinediones and gliptins, are reported to increase the rate of hospitalization for HF during clinical trials (Udell et al., 2015). Therefore, along with DM-HF pathophysiology, mechanistic insight into anti-diabetic drug-induced HF has become essential for cardiovascular safety management of DM patients.

Gliptins are inhibitors of dipeptidyl peptidase 4 (DPP4), a serine peptidase, that degrades glucagon like peptide-1 and in turn augments incretin effect by increasing prandial insulin and reducing glucagon secretion from pancreatic beta and alpha cells, respectively. During the cardiovascular safety trial, TECOS, sitagliptin did not show any effect on the hospitalization of HF in patients suffering from T2DM (Green et al., 2015). However, the SAVOR-TIMI 53 trial reported a 27% increase in the risk of HF in diabetes patients undergoing saxagliptin therapy (Scirica et al., 2013). This clinical observation is supported by our previous data that link saxagliptin to cardiac dysfunction and HF under *in vitro* and *ex vivo* conditions (Koyani et al., 2017). At molecular level, saxagliptin inhibited Ca^2+^/calmodulin-dependent protein kinase II (CaMKII)-mediated phospholamban (PLB) phosphorylation as well as intracellular protein kinase C (PKC) activity in cardiomyocytes.

Apart from DPP4, the DPP4 gene family also includes DPP8 and DPP9 (Yu et al., 2010). DPP8 and DPP9, expressed in various tissues and cells are cytosolic enzymes that have the ability to cleave DPP4 substrates including glucagon-like peptide-1 (Yu et al., 2010). Given the fact that the gliptin-target, DPP4, is not expressed by cardiomyocytes, we set out to explore a potential role of the two cardiomyocyte-resident DPP isoforms, DPP8 and DPP9, in gliptin-mediated signaling events. Despite structural homology to DPP4 (Yu et al., 2010), the role of DPP8/9 in cardiac function is yet unclear. Therefore, we aimed to examine and compare the effects of saxagliptin and sitagliptin on intracellular signaling that may lead to contractile/electrophysiological dysfunction and HF.

## Materials and Methods

### Cell Culture

HL-1 cells (a murine cardiomyocyte cell line, Sigma-Aldrich, Vienna, Austria) were cultured in fibronectin (0.5% [w/v])/gelatin (0.02% [w/v]) coated flasks and maintained in Claycomb medium (Sigma-Aldrich) (Claycomb et al., 1998) containing 10% (v/v) fetal bovine serum (FBS, Thermo Fisher Scientific, IL, United States), 0.1 mM norepinephrine, 2 mM L-glutamine, 100 IU/ml penicillin and 100 μg/ml streptomycin (Sigma-Aldrich), and kept at 37°C under 5% CO_2_, as previously described (Scheruebel et al., 2014).

### Incubation Protocol

HL-1 cells were treated with indicated (in respective Figures and Figure legends) concentrations of saxagliptin or sitagliptin (Adooq Bioscience, CA, United States, dissolved in DMSO, final concentration of DMSO – 0.01% [v/v]) for indicated time periods.

### Cardiomyocyte Isolation and Patch Clamp

Cardiomyocytes were isolated from adult mice (14–18 weeks old, either sex). The experimental procedure and number of used animals were approved by the ethics committee of the Federal Ministry of Science, Research and Economy of the Republic of Austria (BMWF-66.010/0038-V/3b/2018). Mice were euthanized (40 mg/kg ketamine and 10 mg/kg xylazine), hearts were quickly removed and cardiomyocytes were isolated as previously described (Ackers-Johnson et al., 2016) using collagenase 2 and 4 (Worthington Biochemical Corporation, NJ, United States). After isolation cardiomyocytes were kept in standard external solution (containing in mM: NaCl 137, KCl 5.4, CaCl_2_ 1.8, MgCl_2_ 1.1, NaHCO_3_ 2.2, NaH_2_PO_4_ 0.4, HEPES/Na^+^ 10, D(+)-glucose 5.6, pH 7.4 adjusted with NaOH). All experiments were performed on the day of isolation.

Action potentials (APs) were recorded in the whole cell configuration of the patch clamp technique using Axopatch 200B amplifier (Molecular Devices, CA, United States) and the A/D – D/A converters Digidata 1322A (Molecular Devices). To record APs, cardiomyocytes were superfused with the standard external solution at 37°C and pipettes were filled with an internal solution (containing in mM: KCl 110, ATP/K^+^ 4.3, MgCl_2_ 2, CaCl_2_ 1, EGTA 11, HEPES/K^+^ 10, pH 7.4 adjusted with KOH, estimated free [Ca^2+^] < 10^-8^ M). For AP recordings cells were stimulated with minimal suprathreshold current pulses (5 ms) at a frequency of 1 Hz, as previously described (Koyani et al., 2017). In order to exclude any initial transient behavior, first 10 APs were excluded from analysis. Ten consecutive APs were analyzed using LabChart 7.0 (Cardiac action potential analysis module, ADInstruments Ltd., Oxford, United Kingdom).

### Ca^2+^ Transient (CaT) Measurements

After incubation with saxagliptin, sitagliptin or TE-C 5007 at indicated concentration for 4 h, cells were washed twice with the standard external solution and incubated with standard external solution containing 1 μM Fura-2-AM and 1 μM Pluronic F-127 (Thermo Fisher Scientific) for 30 min at 25°C. CaT was assessed by field stimulation (platinum electrode distance: 1 cm; pulse duration: 5 ms; suprathreshold pulse amplitude: 4 V/cm) at a frequency of 1 Hz using a video-based sarcomere length detection system (IonOptix Corporation, MA, United States) at 37°C. Fluorescence intensities were measured at 340 and 380 nm of excitation and at 510 nm of emission wavelengths using a dual excitation light source. The F340/F380 ratio was used as an index of cytosolic Ca^2+^ concentration and to calculate CaT relaxation tau (τ_CaT_). Data were analyzed using Clampfit 10.2 (Molecular Devices) and LabChart 7.0 (peak analysis module, ADInstruments Ltd., Oxford, United Kingdom).

### Fluorescence Microscopy

Fluorescamine (50 mg/ml in acetone, Sigma-Aldrich) and saxagliptin/sitagliptin were mixed in borate buffer (pH 8.5) at indicated concentrations (at 25°C). The reaction mixture was evaporated to dryness and dissolved in the standard external solution (Koyani et al., 2017). HL-1 cells were incubated with saxagliptin/sitagliptin-fluorescamine adduct for 4 min and washed with the standard external solution (see above) at 37°C. Fluorescence images were captured at excitation/emission wavelengths of 390/470 nm.

### Western Blot Analysis

HL-1 cells or mouse left ventricle (LV) were lysed in ice-cold lysis buffer (containing in mM: HEPES 50, NaCl 150, EDTA 1, Na_4_P_2_O_7_ 10, Na_3_VO_4_ 2, NaF 10, 1% [v/v] Triton X-100, 10% [v/v] glycerol, pH 7.4, Sigma-Aldrich) containing a Protease Phosphatase Inhibitor Cocktail (Thermo Fisher Scientific). Pellets were separated by centrifugation at 13,000 rpm (4°C, 10 min). Protein estimation of whole cell was performed using BCA protein assay kit (Thermo Fisher Scientific). Whole cell protein lysates (50 μg) were added to 10 μl of NuPAGE LDS sample buffer (Invitrogen) containing 2 μl of NuPAGE sample reducing agent (Invitrogen). After heating for 10 min at 70°C, proteins were separated by electrophoresis on NuPAGE 4–12% Bis-Tris gel (Invitrogen) and transferred to nitrocellulose membranes (Invitrogen, 0.2 μm) (Jain et al., 2015). Membranes were blocked with 5% (w/v) non-fat milk in Tris-buffered saline containing Tween 20 (TBST, 25°C, 2 h) and incubated with primary antibodies overnight at 4°C. The following primary antibodies (diluted in 5% [w/v] BSA-TBST) were used: phospho-CaMKII (pCaMKII, T286, 1:1000, Abcam, ab32678), phospho-PLB (pPLB, T17, 1:5000, Badrilla, A010-13), DPP8 (1:500, Santa Cruz, sc-376399), DPP9 (1:500, Santa Cruz, sc-271634), CaMKII (1:500, Santa Cruz, sc-9035), and PLB (1:2000, Thermo Fisher Scientific, MA3-922). After washing, the membranes were incubated with HRP-conjugated goat anti-mouse IgG (1:10,000, Cell Signaling, 7076) or goat anti-rabbit IgG (1:10,000, Cell Signaling, 7074) (25°C, 2 h). Immunoreactive bands were visualized using Immobilon Western Chemiluminescent HRP substrate (Merck Millipore, Vienna, Austria) and developed by Bio-Rad ChemiDoc MP Imaging System. For normalization, membranes were stripped with stripping buffer (58.4 g/l NaCl, 7.5 g/l glycine, pH 2.15), blocked and incubated with a primary antibody against glyceraldehyde 3-phosphate dehydrogenase (GAPDH, 1:1000, Santa Cruz, sc-25778). Densitometric evaluation of immunoreactive bands was performed using Image Lab 4.1 software.

### RNA Isolation and Quantitative Real-Time PCR (qPCR)

QIAshredder and RNeasy Mini Kit (Qiagen, Hilden, Germany) were used to isolate RNA from HL-1 cardiomyocytes according to the manufacturer’s protocol. After determining RNA concentration, one μg RNA was reverse transcribed using High-Capacity cDNA Reverse Transcription Kit (Applied Biosystems, Foster City, CA, United States) according to the supplier’s manual. For gene quantification, six ng cDNA per template was used with GoTaq qPCR Master Mix (Promega, Vienna, Austria) and gene specific primers. The qPCR protocol was performed by LightCycler 480 system (Roche Diagnostics, Vienna, Austria). The following gene specific primers from Qiagen were used: GAPDH (QT01658692), DPP8 (QT00143388), and DPP9 (QT00144165). Relative gene expression levels were normalized to GAPDH and calculated using ΔΔCT method (Koyani et al., 2016).

### Gene Silencing

HL-1 cells were transfected with siRNAs (four different constructs) specific for DPP8 or DPP9 (40 nM each, Qiagen), or scrambled negative control siRNA (40 nM si-scr; Allstars negative control siRNA, Qiagen) (Koyani et al., 2014). The siRNA transfection was performed using Lipofectamine 3000 (Invitrogen) according to the supplier’s manual. Briefly, HL-1 (50% confluent) were transfected with 1 ml medium (composition of medium is given above, without FBS and antibiotics) containing 2 μl of Lipofectamine 3000 and the respective siRNA for 6 h at 37°C. Transfection medium was replaced with medium (containing FBS and antibiotics) and cells were grown for another 24 h. The mRNA expression levels of silenced genes were measured using qPCR (see above). In parallel, transfected cells were treated with saxagliptin or sitagliptin to follow protein expression using Western blot (see above) or PKC activity assay (see below).

### PKC Activity Assay

After transfection and/or treatment with saxagliptin, sitagliptin or TC-E 5007, HL-1 cells were washed and lysed in lysis buffer containing Protease Phosphatase Inhibitor Cocktail. Estimation of active PKC was performed using a PKC kinase activity kit (ADI-EKS-420A, Enzo Life Sciences, NY, United States) according to the manufacturer’s protocol.

### Structural Analysis

The 3D structures of DPP4 bound to saxagliptin (PDB code: 3bjm) and sitagliptin (PDB code: 1×70) were aligned based on the 3D structure of DPP4 using pymol 1.8.2. As a result, theoretical models of DPP4 bound to both saxagliptin and sitagliptin were created allowing the direct comparison of the DPP4 amino acids involved in binding of gliptins. This 3D model was subsequently aligned with either the 3D structure of DPP8 (PDB code: 6eoo) or DDP9 (PDB code: 6eoq) based on structural similarity between DPP4 and DPP8/9 using pymol 1.8.2. As a result, theoretical models of DPP8 and DPP9 bound to both saxagliptin and sitagliptin were created. The amino acids of DPP8/9 present in the gliptin binding interface were then compared to those of DDP4 in order to access the potential for sitagliptin- and/or saxagliptin-binding to DPP8/9.

### Statistical Analysis

Statistical analyses were performed using IBM SPSS Statistics 23 software. Approximate normal distribution of data was assessed by visual (histograms and normal Q-Q plots) and numerical investigation (*z*-value of skewness and kurtosis; *p*-value of Shapiro–Wilk test). After checking homogeneity of variance by Levene’s test, between groups comparisons were evaluated by unpaired Student’s *t*-test, one-way ANOVA (followed by Tukey’s *post hoc* test) or ANCOVA to compare the effect of saxagliptin/sitagliptin vs. rundown by adjusting the matched control values (covariates). ANCOVA was only applied when covariates and regression slopes were not different between the compared groups. *P*-values ≤ 0.05 were considered statistically significant. All tests were 2-sided.

## Results

### Fluorescamine-Derivatives of Saxagliptin and Sitagliptin Are Internalized by Cardiomyocytes

As saxagliptin is internalized by cardiomyocytes (Koyani et al., 2017), we aimed to investigate whether sitagliptin is also internalized by cardiomyocytes. For these experiments, both compounds were coupled with fluorescamine (a non-fluorescent dye that yields fluorescence upon reaction with primary amines in a 1:1 stoichiometry) in order to visualize cellular uptake and localization.

**FIGURE 1:**
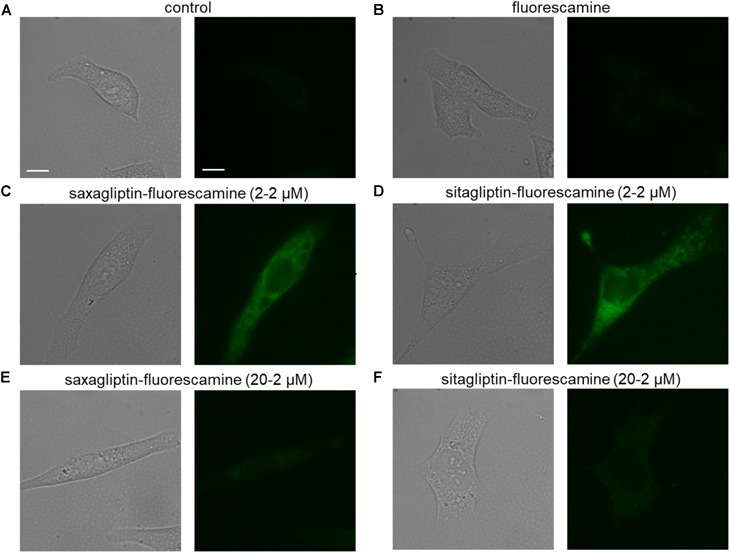
Saxagliptin and sitagliptin internalizes into cardiomyocytes. Representative bright-field (left panels, **A–F**) and corresponding fluorescent images (right panels, **A–F**) of HL-1 cardiomyocytes treated with standard external solution **(A)** alone (control) or containing **(B)** fluorescamine (2 μM), saxagliptin-fluorescamine adduct [**(C)** 2–2 μM or **(E)** 20–2 μM] or sitagliptin-fluorescamine adduct [**(D)** 2–2 μM or **(F)** 20–2 μM] for 5 min (*n* = 6, scale bar: 10 μm). “n” represents the number of experiments.

Untreated (Figure 1A) or fluorescamine-treated (Figure 1B) HL-1 cells showed no fluorescence (unbound fluorescamine has a very short half-life (5–10 s) in an aqueous solution; Udenfriend et al., 1972), while incubation with the saxagliptin-fluorescamine (2–2 μM) complex showed pronounced fluorescence that is located exclusively in the cytosol (Figure 1C). A similar intracellular fluorescence pattern was observed in cells incubated with the sitagliptin-fluorescamine complex (Figure 1D). To determine specificity of drug uptake, we added a 10-fold molar excess (20–2 μM) of unlabeled saxagliptin/sitagliptin to investigate competition between free and fluorescamine-bound drugs. Under these conditions, fluorescence intensity was reduced almost to basal level (Figures 1E,F). These data indicate that sitagliptin is internalized by cardiomyocytes and localizes in the cytosol, similarly, as reported for saxagliptin (Koyani et al., 2017).

### Saxagliptin but Not Sitagliptin Inhibits the CaMKII-PLB Axis

**FIGURE 2:**
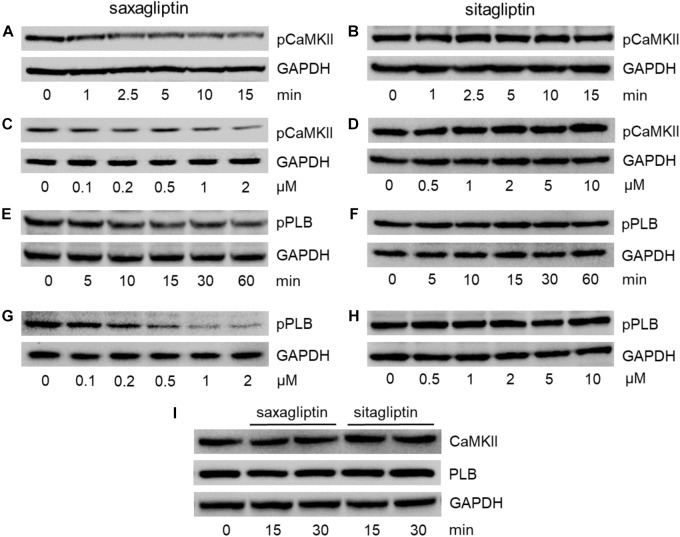
Saxagliptin but not sitagliptin inhibits the CaMKII-PLB axis. Representative Western blots (*n* = 6) for **(A–D)** pCaMKII (T286), **(E–H)** pPLB (T17), and **(I)** CaMKII and PLB expression in HL-1 cardiomyocytes treated with saxagliptin **(A,C,E,G)** or sitagliptin **(B,D,F,H)** for the indicated time points (**A**,**B**,**E**,**F,I**, 2 μM) and concentrations (**C,D**, 5 min; **G,H**, 30 min). GAPDH was used as a loading control. “n” represents the number of experiments.

Since both gliptins are internalized by cardiomyocytes (Figures 1C,D) and saxagliptin was reported to attenuate phosphorylation of CaMKII and PLB, we aimed to evaluate whether sitagliptin displays similar pharmacological effects on the CaMKII-PLB axis. In line with our previous data (Koyani et al., 2017), incubation of HL-1 cells with 2 μM saxagliptin resulted in reduced CaMKII phosphorylation (T286) starting from 2.5 min (Figure 2A). In contrast, 2 μM sitagliptin failed to inhibit pCaMKII expression (Figure 2B). Concentration-dependent experiments demonstrate that saxagliptin (but not sitagliptin) inhibits CaMKII phosphorylation starting from 200 nM (Figures 2C,D, respectively). Additionally, sitagliptin treatment (up to 10 μM) had no effect on the CaMKII phosphorylation (Figure 2D).

As phosphorylation of CaMKII promotes PLB phosphorylation (T17), we examined pPLB expression level. Treatment of HL-1 cardiomyocytes with saxagliptin resulted in reduced immunoreactive pPLB band starting from 15 min (Figure 2E). In parallel, concentration-dependent experiments show saxagliptin-reduced pPLB expression starting from 200 nM dose (Figure 2G). Conversely, neither time- (Figure 2F) nor concentration-dependent experiments (Figure 2H) showed an effect of sitagliptin on PLB phosphorylation levels. Moreover, total CaMKII or PLB levels did not change in response to gliptin treatment (Figure 2I). Densitometric evaluation of Western blots and statistical analysis is shown in Supplementary Figures 1–3.

### Structural Analysis of Gliptins-DPP4/8/9 Interaction and Effect of DPP8/9 Inhibition

**FIGURE 3:**
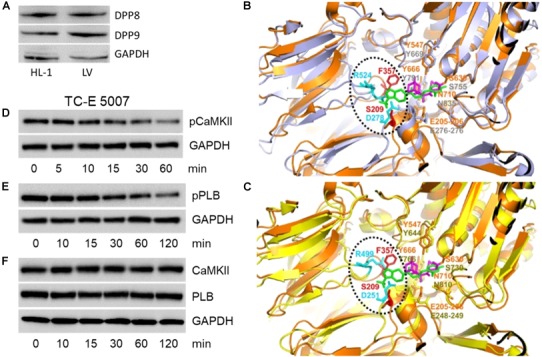
Structural analysis of gliptins-DPP4/8/9 binding. **(A)** A representative Western blot (*n* = 3) showing expression of DPP8 and DPP9 in total protein lysate (50 μg) of HL-1 cardiomyocytes and mouse LV. **(B)** 3D structural alignment of saxagliptin bound to DPP4 (PDB code: 3bjm) and sitagliptin (PDB code: 1×70) aligned with the 3D structure of DPP8 (PDB code: 6eoo) using pymol. The 3D structure of DPP4 and DPP8 are shown as cartoon representation and colored in orange and gray, respectively. The DPP4-bound forms of saxagliptin and sitagliptin are shown in sticks and colored in magenta and green, respectively. The DPP4 and DPP8 amino-acids present in the gliptin-binding surface of DPP4 are shown in sticks and colored in orange and gray, respectively, if conserved in the structural model or, in red and cyan, if not. **(C)** 3D structural alignment as described in **(B)** but using DPP9 (PDB code: 6eoq, colored in yellow) in place of DPP8. Representative Western blots (*n* = 6) for **(D)** pCaMKII (T286), **(E)** pPLB (T17), and **(F)** CaMKII and PLB expression in HL-1 cardiomyocytes treated with TC-E 5007 (2 μM) for the indicated time points. GAPDH was used as a loading control. “n” represents the number of experiments.

The primary target of gliptins, DPP4, is not expressed by cardiomyocytes (Shigeta et al., 2012; Koyani et al., 2017). However, cytosolic localization of gliptins (Figures 1C,D) may lead to their interaction with the cardiomyocyte resident DPP isoforms (DPP8 and DPP9) due to their structural homology with DPP4. To test this hypothesis, we first investigated the expression of DPP8/9 in HL-1 cells and mouse LV by Western blotting. Figure 3A confirms the presence of DPP8/9 in HL-1 cells and mouse LV.

Based on the three-dimensional structure of DPP4 bound to saxagliptin (PDB code: 3bjm; Metzler et al., 2008) and sitagliptin (PDB code: 1×70; Kim et al., 2005) we identified key amino acids of DPP4 that are involved in gliptin binding (Figures 3B,C). Close inspection of the interaction modes shows that DPP4-saxagliptin interaction motif (S630, Y547) is conserved in DPP8 (S755, Y669) and DPP9 (S730, Y644). However, the region around DPP4-sitagliptin binding comprises of amino acids (S209, F357) that are neither conserved in DPP8 nor in DPP9. Additionally, DPP8 and DPP9 harbor two charged amino-acids in the surrounding of F357 (D278/R524 for DPP8 and D251/R499 for DPP9) that might interfere with binding of sitagliptin to DPP8/9 either via steric clashes and/or charge repulsion. These structural analyses suggest that binding of saxagliptin is conserved in DPP8/9 whereas binding of sitagliptin is weakened or even abolished.

To investigate the role of DPP8/9 in saxagliptin-induced intracellular signaling, we used TC-E 5007, a DPP8/9 inhibitor with IC_50_ of 145 and 242 nM, respectively (Lankas et al., 2005). Treatment of HL-1 cardiomyocytes with 2 μM TC-E 5007 resulted in a time-dependent inhibition of pCaMKII (T286) and pPLB (T17) expression starting from 30 and 60 min, respectively (Figures 3D,E). In contrast, TC-E 5007 did not affect total CaMKII and PLB protein levels (Figure 3F). Densitometric evaluation of blots and statistical analysis is shown in Supplementary Figure 4.

### Saxagliptin Inhibits the CaMKII-PLB Axis Dependent on DPP9

**FIGURE 4:**
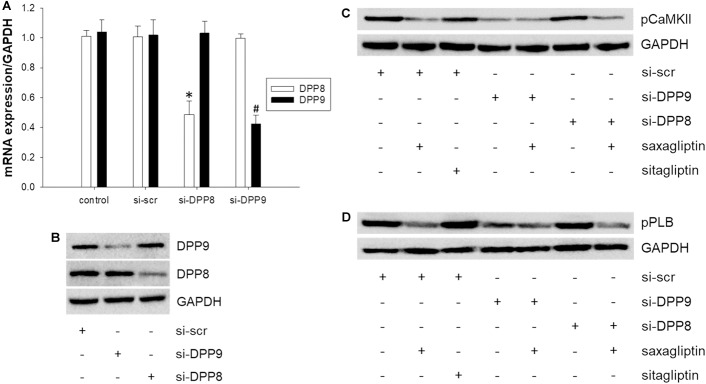
Saxagliptin inhibits the CaMKII-PLB axis via DPP9 inhibition. **(A)** mRNA and **(B)** protein expression of DPP8 and DPP9 in HL-1 cardiomyocytes transfected with si-scr, si-DPP8 or si-DPP9. GAPDH was used as a house keeping gene/protein. A representative Western blot showing **(C)** pCaMKII (T286), and **(D)** pPLB (T17) expression in HL-1 cardiomyocytes transfected with si-DPP8 or si-DPP9, and/or treated with saxagliptin or sitagliptin (2 μM each) as indicated for **(C)** 5 min and **(D)** 30 min. All values are expressed as mean ± SEM (*n* = 6). ^∗^*p* < 0.05 vs. si-scr DPP8 and ^#^*p* < 0.05 vs. si-scr DPP9 by one-way ANOVA followed by Tukey’s *post hoc* test. “n” represents the number of experiments.

To further clarify the individual role(s) of DPP8 and/or DPP9 in saxagliptin-reduced pCaMKII and pPLB expression in cardiomyocytes, a RNA interference approach was used. HL-1 cells were transfected with specific siRNA against DPP8 or DPP9. qPCR analyses revealed that siRNA transfection significantly reduced mRNA levels of the target DPP isoform (∼52% of DPP8 and ∼58% of DPP9) without affecting expression of the non-target DPP isoform (Figure 4A). In contrast, at protein level we observed ∼64 and 79% reduction of DPP8 and DPP9 expression, respectively (Figure 4B). There was no difference in the expression of DPP8/9 between non-transfected and si-scr transfected cells (Figures 4A,B).

Next, we evaluated the effect of DPP knock-down on phosphorylation of CaMKII and PLB. Treatment of control cardiomyocytes with saxagliptin (but not sitagliptin) reduced pCaMKII expression (Figure 4C). Furthermore, silencing of DPP9 (but not DPP8) resulted in reduction of CaMKII phosphorylation in a similar manner as observed with saxagliptin alone. The efficacy of saxagliptin to inhibit CaMKII phosphorylation is blunted in cardiomyocytes transfected with si-DPP9. Conversely, saxagliptin effectively inhibited pCaMKII expression in DPP8 knocked down cardiomyocytes.

Further, we tested these conditions for PLB phosphorylation. Similar to CaMKII phosphorylation, pPLB expression was dampened by saxagliptin (but not sitagliptin) and DPP9 (but not DPP8) knock-down (Figure 4D). In line, the efficacy of saxagliptin was abolished in DPP9 (but not DPP8) silenced cells (Figure 4D). Densitometric evaluation of blots and statistical analysis is shown in Supplementary Figure 5. These data demonstrate that saxagliptin targets DPP9 and thereby impairs the CaMKII-PLB axis in cardiomyocytes.

**FIGURE 5:**
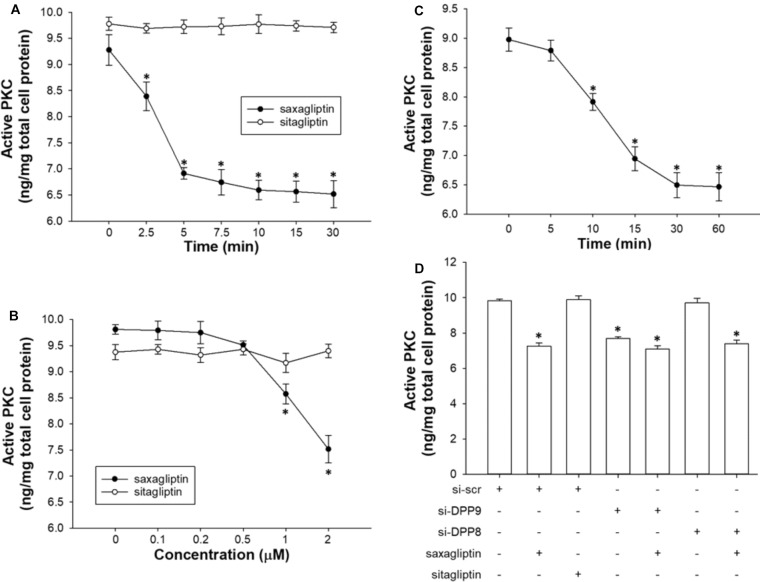
Saxagliptin inhibits PKC via DPP9 inhibition. Quantification of active PKC levels in HL-1 cardiomyocytes treated with saxagliptin or sitagliptin in a **(A)** time-dependent (2 μM) or **(B)** concentration-dependent manner (10 min), and **(C)** TC-E 5007 (2 μM) for indicated time periods. **(D)** HL-1 cardiomyocytes were transfected with si-DPP8 or si-DPP9, and/or treated with saxagliptin or sitagliptin (2 μM) for 10 min as indicated to follow measurements of active PKC. All values are expressed as mean ± SEM (*n* = 6). ^∗^*p* < 0.05 vs. **(A,C)** 0 min, **(B)** 0 μM, and **(D)** si-scr by one-way ANOVA followed by Tukey’s *post hoc* test. “n” represents the number of experiments.

### Saxagliptin Inhibits PKC Activity, Dependent on DPP9

As reported previously (Koyani et al., 2017) and here (Figures 5A,B), saxagliptin significantly reduced PKC activity in cardiomyocytes in a time- (from 2.5 min) and concentration-dependent manner (from 1 μM). In contrast, our present data show that the treatment of cardiomyocytes with sitagliptin had no effect on intracellular PKC activity (Figures 5A,B). In parallel, the DPP8/9 inhibitor TC-E 5007 reduced active PKC levels in a time-dependent manner starting from 10 min in HL-1 cardiomyocytes (Figure 5C).

Next, we silenced DPP8/9 expression to get an indication whether one or both DPP isoforms might play a role in saxagliptin-reduced PKC activity. Ablation of DPP9 resulted in decreased PKC activity, whereas si-DPP8 had no effect (Figure 5D). The efficacy of saxagliptin was significantly abolished in DPP9 (but not DPP8) silenced cardiomyocytes. These data reveal that DPP9 links saxagliptin with reduced PKC activity in cardiomyocytes.

### Saxagliptin and TC-E 5007 Impairs CaT Relaxation and Prolongs APD

**FIGURE 6:**
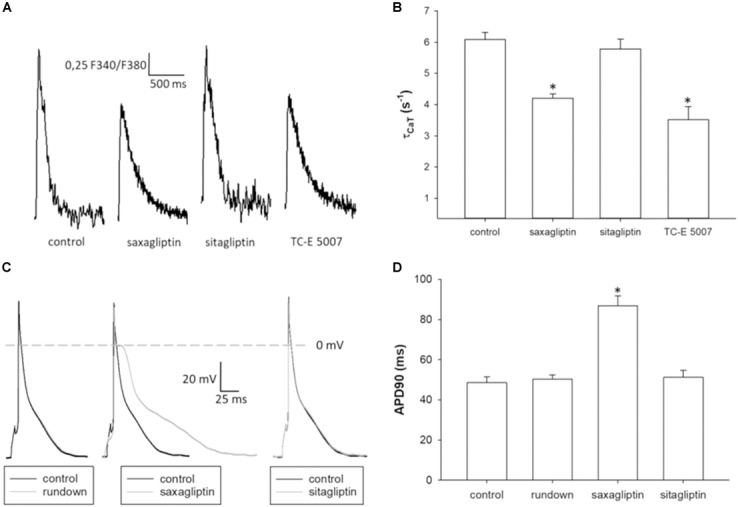
Saxagliptin and TC-E 5007 impairs CaT relaxation and prolongs APD. **(A)** Representative CaT traces and **(B)** CaT decay tau (τ_CaT_) of control and a saxagliptin-, sitagliptin- or TC-E 5007 (2 μM and 2 h for all)-treated mouse ventricular cardiomyocytes stimulated at 1 Hz frequency (*n* = 14). **(C)** Representative action potentials (AP) and **(D)** AP duration at 90% repolarization of mouse ventricular cardiomyocytes before (control) and after 5 min superfusion with either external solution alone (rundown), saxagliptin or sitagliptin (2 μM for both) and stimulated at 1 Hz frequency (*n* = 10/5). Controls of rundown, saxagliptin and sitagliptin groups are combined to improve readability of the figure **(D)**. All values are expressed as mean ± SEM. “n” represents the number of **(B)** experiments and **(C)** cardiomyocytes/animals. ^∗^*p* < 0.05 vs. control by **(B)** one-way repeated-measure ANOVA followed by Tukey’s *post hoc* test or **(D)** ANCOVA.

To evaluate the effects of saxagliptin- and TC-E 5007-evoked intracellular signaling on cardiomyocyte function, we performed CaT and patch clamp analysis. Saxagliptin and TC-E 5007 impaired the CaMKII-PLB axis that regulates sarcoendoplasmic reticulum (SR) Ca^2+^-ATPase 2a (SERCA2a) function in Ca^2+^ removal during cardiac relaxation. Therefore, reciprocal of τ_CaT_ was analyzed to assess cardiac relaxation. Treatment of mouse ventricular cardiomyocytes with saxagliptin and TC-E 5007 resulted in impaired cardiac relaxation, whereby sitagliptin was ineffective (Figures 6A,B). In parallel, electrophysiological analysis showed prolongation of APD upon treatment with saxagliptin but not sitagliptin (Figures 6C,D).

## Discussion

**FIGURE 7:**
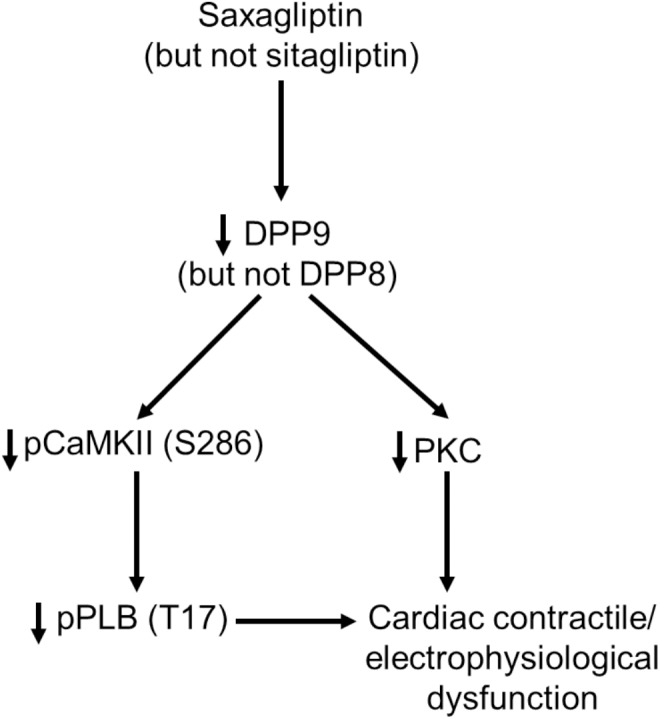
A schematic presentation of the observed DPP9-mediated effects of saxagliptin on cardiac signaling, contractility and electrophysiology.

In the present study we identified DPP9 as a primary target of saxagliptin that in turn initiates intracellular signaling contributing to cardiac contractile and electrophysiological dysfunction (Figure 7). Both, saxagliptin and sitagliptin internalize into cardiomyocytes and localize exclusively in the cytosol. Basically, the primary target of gliptins, DPP4, is not expressed by cardiomyocytes. However, cardiomyocytes do express other DPP isoforms, namely DPP8 and DPP9. Structural analysis reveals favorable interaction of saxagliptin (but not sitagliptin) with both the DPP isoforms. Moreover, saxagliptin (but not sitagliptin) treatment resulted in reduced phosphorylation of CaMKII and PLB, and PKC activity. These results were recapitulated while using a DPP8/9 inhibitor, TC-E 5007. In contrast, these effects were observed only in DPP9 (but not DPP8) silenced cardiomyocytes. Furthermore, the efficacy of saxagliptin to inhibit the CaMKII-PLB axis and PKC was abolished in cardiomyocytes transfected with si-DPP9 (but not si-DPP8). At functional level, saxagliptin and TC-E 5007 impaired CaT relaxation and saxagliptin prolonged APD of mouse ventricular cardiomyocytes.

Although gliptins have high aqueous solubility, *in vitro* studies show a significant membrane permeability of saxagliptin (Koyani et al., 2017), sitagliptin (Lee et al., 2016) as well as vildagliptin (He et al., 2009). Lee et al. (2016) have reported that sitagliptin permeated endothelial membrane in a dose-dependent manner and activates the Src/VE-cadherin pathway. This intracellular cascade was directly linked to vascular leakage in the retina (Lee et al., 2016). Internalization of saxagliptin into cardiomyocytes was further reported to inhibit cardiac kinases including CaMKII and PKC that lead to cardiac dysfunction (Koyani et al., 2017). Like saxagliptin, we observed that fluorescamine-bound sitagliptin is internalized by cardiomyocytes and localized in the cytosol.

DPP8 and DPP9 are primarily localized in the cytosol and nucleus in T-cells (Ross et al., 2018). Despite structural similarities and cellular localization, increasing evidence suggests distinct roles of both DPP isoforms. We report that both DPPs are expressed in HL-1 cardiomyocytes and mouse LV. A previous study showed high expression of DPP9 in human heart using Northern blot analysis (Olsen and Wagtmann, 2002). Although functions of these DPPs in cardiac signaling are largely unknown, both DPPs play a role in monocytes/macrophage pyroptosis (Okondo et al., 2017) as well as in leukemia (Spagnuolo et al., 2013). Overexpression of DPP9 correlates with poor 5-year overall survival in patients with non-small cell lung cancer (Tang et al., 2017).

Here, we show a specific role of DPP9 in cardiac signaling where knock-down of DPP9 resulted in inhibition of the CaMKII-PLB axis and PKC activity. Although the exact mechanism of DPP9-induced cardiac signaling remains to be elucidated, a previous report addresses a potential role of protein phosphatase 2A (PP2A) in the observed effects (Zhang et al., 2015). Zhang and coworkers have reported phosphatase 2A inhibitor (I2PP2A) as a substrate of DPP9 (Zhang et al., 2015). Moreover, PP2A is known to regulate both CaMKII (Erickson, 2014; Lubbers and Mohler, 2016) and PKC (Sontag et al., 1997; Boudreau et al., 2002). Therefore, one may predict a plausible role of PP2A in DPP9-mediated regulation of CaMKII and PKC in cardiac signaling. However, further studies are required to confirm these signaling events.

Although gliptins are developed in order to inhibit activity of DPP4 specifically, partial inhibition of DPP8/9 by gliptins occurs due to the structural homology of these DPP isoforms. Structural analysis reveals that amino acids involved in saxagliptin-DPP4 binding are also conserved in DPP8/9 (Figure 3B). Apparently, the triazolopyrazine moiety with the trifluoromethyl substituent of sitagliptin represents the “anchor lock domain” of this inhibitor. The anchor lock domain was shown to tightly bind to the S2 extensive subsite in DPP4 (Berger et al., 2018). Most importantly, the triazolopyrazine group binds to F357 in the S2 extensive subsite of DPP4 and this residue may apparently facilitate high affinity binding of sitagliptin. Additionally, two charged amino acids present in DPP8/9 (Figure 3C) may repel the binding of sitagliptin to DPP8/9. These observations obtained from molecular modeling in the present study are in line with *in vitro* inhibition of DPP8/9 by gliptins. The K_i_ values for inhibition of human DPP8/9 by saxagliptin are 508/98 nM and those for sitagliptin are 33,780/55,142 nM, respectively (Wang et al., 2012). Based on these data (Wang et al., 2012) and the present structural analysis, it is likely to assume an inhibition of DPP8/9 by saxagliptin (but not sitagliptin) under *in vivo* conditions. Moreover, the observed effects of saxagliptin on the CaMKII-PLB axis and PKC activity are dependent on DPP9. Saxagliptin inhibits DPP9 *in vitro* at K_i_ of 98 nM (Wang et al., 2012), a concentration that is relevant to the plasma C_max_ of saxagliptin following a 5 mg single dose (24 ng/ml, equivalent to ∼76 nM) (Onglyza, 2016).

CaMKII is one of the major multifunctional protein kinases that contribute to the development and progression of HF. Increasing evidence suggests that aberrant CaMKII signaling is a core mechanism in HF and related arrhythmias (Hasegawa et al., 2016). CaMKII regulates function of ion channels involved in cardiac electrophysiology and Ca^2+^ homeostasis. The latter phenomenon includes PLB that is regulated by CaMKII-mediated phosphorylation. Upon dephosphorylation, PLB inhibits SERCA2a, which pumps ∼70% of cytosolic Ca^2+^ into SR during cardiac relaxation (Bers, 2000). Saxagliptin-reduced PLB phosphorylation resulted in reduced Ca^2+^ removal by SERCA2a as well as Na^+^-Ca^2+^ exchanger (Koyani et al., 2017). These effects were supported by elevated end-diastolic LV pressure and reduced end-systolic LV pressure. One or both of these parameters are observed in failing hearts. Our present data show that saxagliptin interferes with the CaMKII-PLB axis in a DPP9-dependent manner, while sitagliptin had no effect on these signaling events. Furthermore, saxagliptin (but not sitagliptin) and TC-E 5007 impaired τ_CaT_ in mouse ventricular cardiomyocytes.

Apart from contractile dysfunction, prolonged APD and QT interval are considered hallmarks of HF (Wang and Hill, 2010). In the present study, we observed prolongation of APD in mouse ventricular cardiomyocytes after superfusion with saxagliptin (but not sitagliptin). Reduced PKC activity, as observed with saxagliptin, was correlated to impaired delayed-rectifier K^+^ current, prolonged APD and QT interval (Koyani et al., 2017). In the present study, we further discovered that DPP9 is involved in saxagliptin-reduced PKC activity. On the contrary, sitagliptin treatment had no effect on PKC activity in cardiomyocytes. This observation is in line with a previous study where sitagliptin was ineffective to PKC activity in human endothelial cells (Hasegawa et al., 2016). However, in THP-1 macrophages, saxagliptin inhibited oxidized low-density lipoprotein-induced pPKC levels (Dai et al., 2014), indicating a link between saxagliptin and PKC pathway.

Our *in vitro* data demonstrating ineffectiveness of sitagliptin on cardiac signaling, contractility and electrophysiology may provide a molecular mechanism for *in vivo* data showing no link between sitaglitpin treatment and risk of HF (Green et al., 2015). Moreover, this is the first study to reveal the role of DPP9 in cardiac signaling, contractility and electrophysiology. Our data reveal DPP9 as a novel saxagliptin-target that initiates intracellular signaling cascades leading cardiac contractile and electrophysiological dysfunction.

### Limitations

The DPP8/9 inhibitor, TC-E 5007, has a slow onset of action on PKC inhibition (10 min, Figure 5C) that lead to prolongation of APD, and we have performed patch clamp experiments in a “before and after superfusion” setting. Therefore, we could not perform patch clamp experiments using TC-E 5007 according to our experimental setting. Moreover, adult primary mouse cardiomyocytes are notoriously hard to keep in culture for prolonged periods of time. This precluded silencing experiments with sufficient knockdown efficiency and cell viability.

## Author Contributions

CK, CT, SS, SK, and IP performed experiments and analyzed the data. BB and TM performed structural analysis. All authors interpreted and discussed data, and contributed to manuscript writing.

## Conflict of Interest Statement

The authors declare that the research was conducted in the absence of any commercial or financial relationships that could be construed as a potential conflict of interest.
