# Supplementary material for: Saxagliptin but Not Sitagliptin Inhibits CaMKII and PKC via DPP9 Inhibition in Cardiomyocytes
Source: Front Physiol. 2018 Nov 14;9:1622. doi: 10.3389/fphys.2018.01622 (PMC6246635; doi:10.3389/fphys.2018.01622)
Supplement: Supplementary file 1 [file Data_Sheet_1.docx]

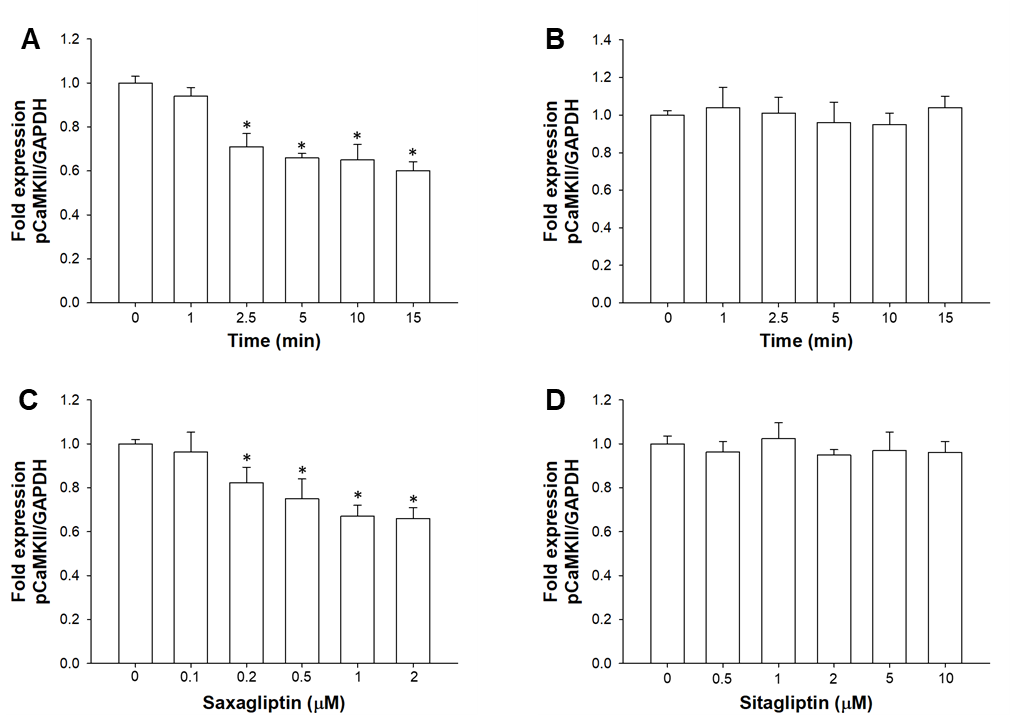


**Supplementary Figure 1**

Densitometric evaluation of immunoreactive bands of Western blots shown in Figure 2A-D, respectively. All values are expressed as mean ± SEM (n=6). *p<0.05 vs. (**A**) 0 min and (**C**) 0 µM by one-way ANOVA followed by Tukey’s post-hoc test.


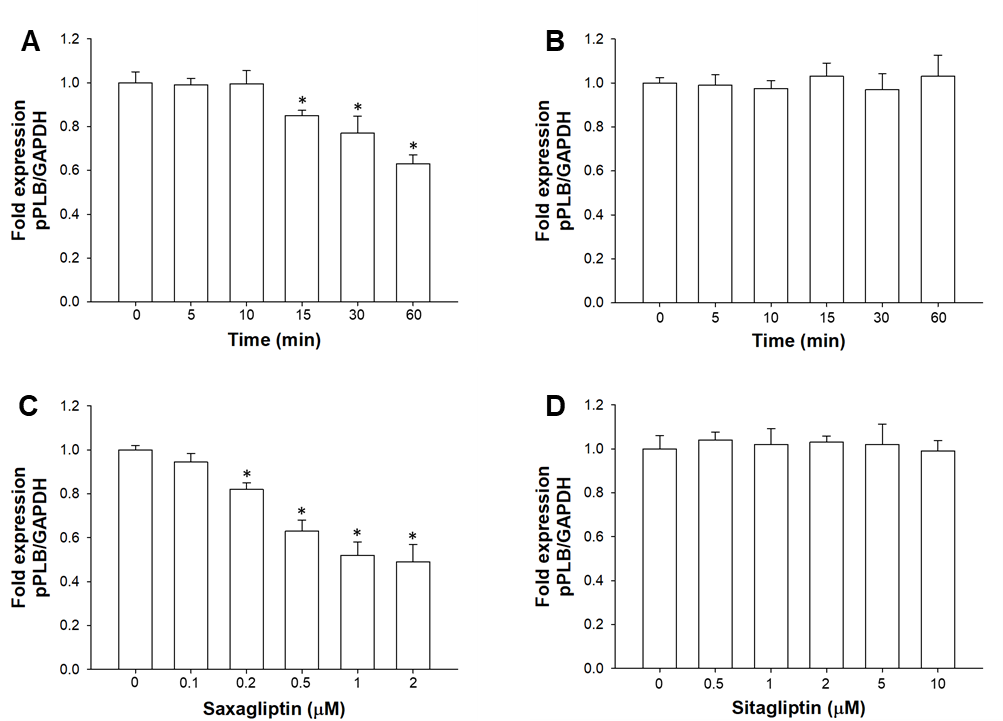


**Supplementary Figure 2**

Densitometric evaluation of immunoreactive bands of Western blots shown in Figure 2E-H, respectively. All values are expressed as mean ± SEM (n=6). *p<0.05 vs. (**A**) 0 min and (**C**) 0 µM by one-way ANOVA followed by Tukey’s post-hoc test.


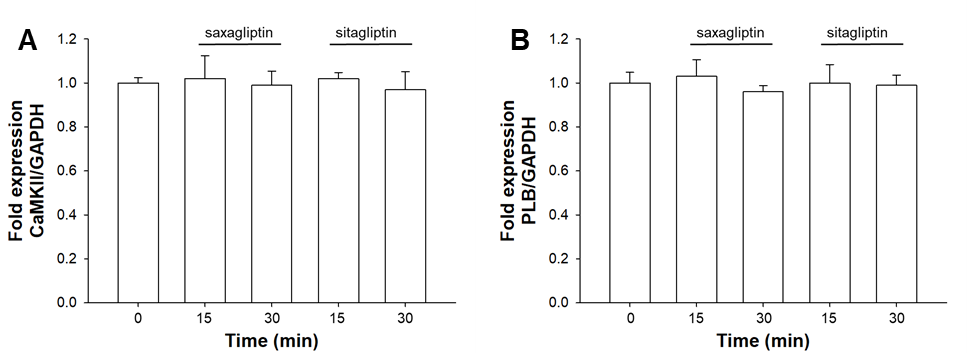


**Supplementary Figure 3**

Densitometric evaluation of immunoreactive bands of Western blots shown in Figure 2I. All values are expressed as mean ± SEM (n=6).


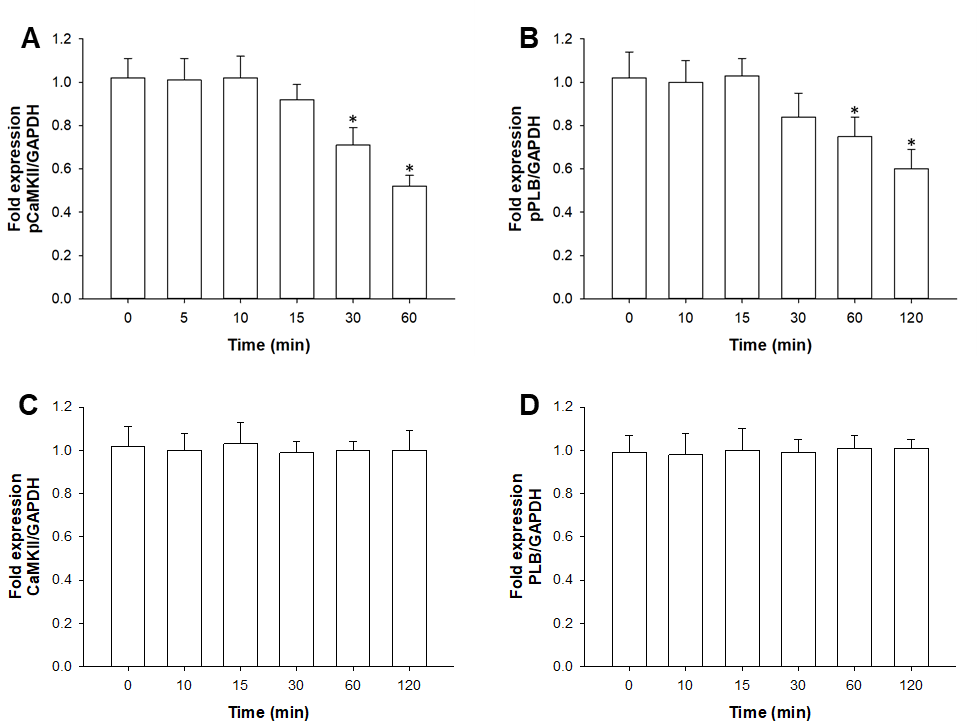


**Supplementary Figure 4**

Densitometric evaluation of immunoreactive bands of Western blots shown in Figure 3D-F, respectively. All values are expressed as mean ± SEM (n=6). *p<0.05 vs. 0 min by one-way ANOVA followed by Tukey’s post-hoc test.


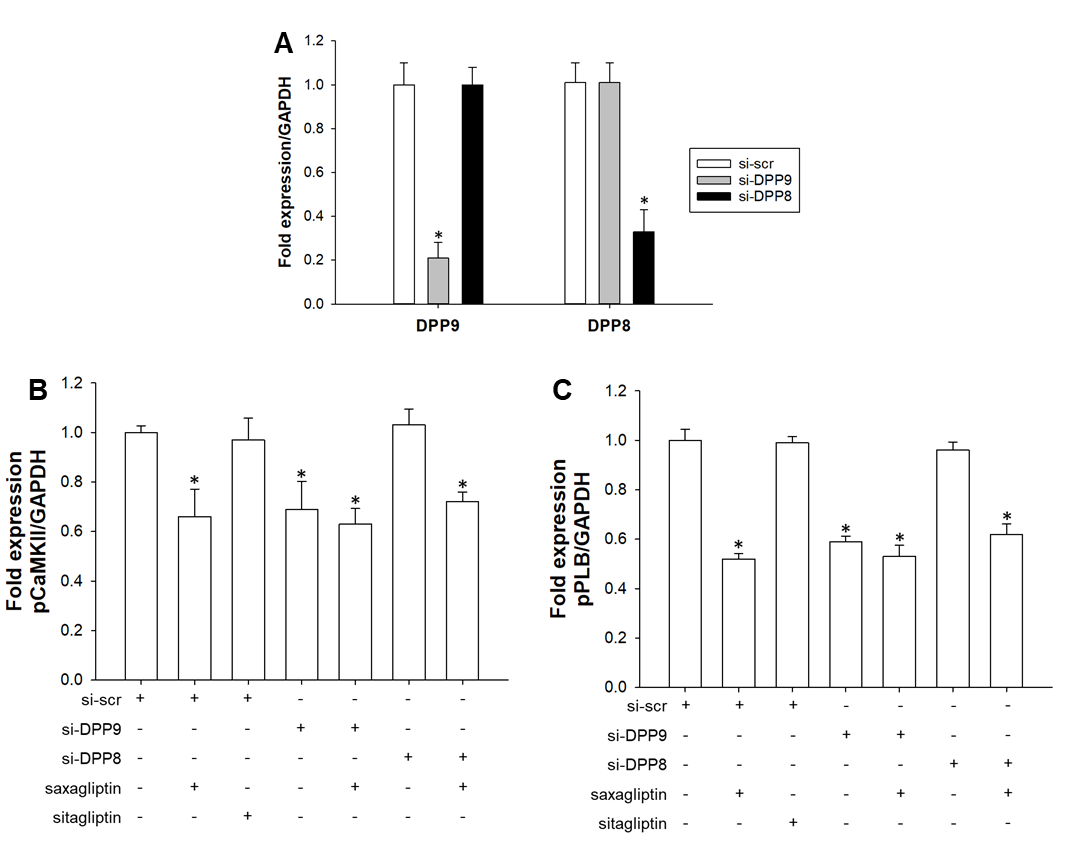


**Supplementary Figure 5**

Densitometric evaluation of immunoreactive bands of Western blots shown in Figure 4B-D, respectively. All values are expressed as mean ± SEM (n=6). *p<0.05 vs. si-scr by one-way ANOVA followed by Tukey’s post-hoc test.
